# Chronic Stress and Stroke Among the Adult Population in the United States

**DOI:** 10.7759/cureus.85363

**Published:** 2025-06-04

**Authors:** Luba Nakhutina, Samy I McFarlane

**Affiliations:** 1 Department of Neurology, State University of New York System (SUNY) Downstate Health Sciences University, Brooklyn, USA; 2 Department of Medicine, State University of New York System (SUNY) Downstate Health Sciences University, Brooklyn, USA

**Keywords:** cardiovascular disease, diabetes, hypertension, obesity, psychological stress, race, sex, s: obesity paradox, stroke, the kessler psychological distress scale (k6)

## Abstract

Objective: This study aimed to examine the association of chronic psychological distress and stroke among a large sample of the adult population in the United States.

Methodology: We utilized data from the National Health Interview Survey (NHIS) spanning 10 years (2004-2013). Stroke history was self-reported, as were other stroke risk factors. Psychological distress was measured using the Six-Item Kessler Psychological Distress Scale (a score of ≥13 indicated distress). We used a logistic regression model to examine the strength of the association between psychological distress and stroke. We used the stepwise approach adjusting for age, body mass index (BMI), and sociodemographic and other major risk factors of stroke, such as hypertension, diabetes, hypercholesterolemia, and smoking.

Results: Among the 284497 individuals who participated in the NHIS and fully completed the Six-Item Kessler Psychological Distress Scale, 158665 (55.7%) were women, and 125832 (44.3%) were men. Prevalence of chronic psychological distress was higher among women compared to men (n=6622 (4.2%) vs. n=3596 (2.9%), respectively; p<0.01). Unadjusted odds ratio for stroke among psychologically distressed individuals was 3.1 (95% CI 2.8-3.3; p<0.01). After adjusting for age, hypertension, diabetes, BMI, hypercholesterolemia, and smoking, the odds of stroke in those with psychological distress was nearly threefold (OR 2.7; 95% CI 2.3-3.2; p<0.01). In our logistic regression model, hypertension conferred a high risk of stroke with an OR of 2.6 (95% CI 2.3-2.8; p<0.01). Diabetes increased the odds of stroke with an OR of 1.9 (95% CI 1.8-2.1; p<0.01). Hypercholesterolemia was also strongly associated with stroke risk (OR 1.5; 95% CI 1.3-1.6). Compared to never smoking, current smokers and former smokers had an OR of 1.99 (95% CI 1.7-2.2; p<0.01) and 1.3 (95% CI 1.2-1.4; p<0.01), respectively. In our model, BMI ≥25 conferred a significant risk of stroke (OR 1.28; 95% CI 1.20-1.35; p<0.01); however, after adjusting for cerebrovascular risk factors, including diabetes, hypertension, and high cholesterol, the stroke odds by obesity reversed (OR 0.86; 95% CI 0.75-0.98; p=0.03). While these stroke risk factors attenuated the association between psychological distress and stroke, the relationship remained highly significant in our final model with additional adjustment for sociodemographic factors, where the OR for stroke was 2.17 (95% CI 1.81.3-2.60; p<0.01).

Conclusion: Our study results demonstrate that psychological distress increases, more than twice, the odds of stroke among the adult population in the United States. Behavioral interventions targeting psychological stress may result in lowering the risk of stroke, as well as of other vascular diseases.

## Introduction

Stroke remains to be one of the top 10 causes of mortality in the United States, with recent data from the Centers for Disease Control and Prevention (CDC) for 2023 showing that stroke is the fourth leading cause among the top 10 causes of death in the United States, after heart disease, cancer, and unintentional death [1]. Furthermore, the associated morbidity and healthcare cost of stroke continue to rise, representing a serious public health concern. A recent retrospective study using data from the past two decades (2000-2020) showed that while there is a favorable outcome trend in stroke mortality, there has been an astronomical increase in the cost of care for the increasing number of surviving stroke victims [2]. These data indicate an exponential growth pattern, forecast per-patient charges for the next 10 years, and demonstrate a cost of USD 287836 by 2030. The authors of the study conclude that per-patient charges increased more than sixfold, with a national bill almost equal to the annual Medicare budget [2]. The authors also recommended a more vigorous approach to stroke prevention, including mitigating stroke risk factors [2].

Risk factors for stroke include modifiable and non-modifiable ones [3-6], where age, sex, race, ethnicity, and genetics are the major non-modifiable risk factors, whereas hypertension, smoking, diabetes, hypercholesterolemia, central obesity, as well as physical inactivity are among the potentially modifiable risk factors, according to the American Heart Association [3]. Hypertension is the major risk factor, particularly when combined with type 2 diabetes and metabolic syndrome [4,5]. Control of cardiovascular risk factors has proven to be quite challenging, particularly among minority populations, with work by our group and other academic medical centers indicating that hypertension, diabetes, and other cardiovascular risk factors were quite suboptimal, with evidence of racial and gender disparity in care [7-10]. Since cardiovascular risk factors are potentially modifiable risks for stroke, identifying these and other potentially modifiable targets for stroke prevention is quite important. Psychological distress appears to be an attractive candidate as a potentially modifiable risk factor for stroke.

Previous studies that have examined the association of psychological stress and cerebrovascular disease or stroke were conducted primarily outside of the United States [11-13], such as Australia [12], China [13], and the United Kingdom [11]. This limits the generalizability of findings for the United States in the context of known geographical and ethnic differences in stroke incidence. Furthermore, some of these studies used different tools in assessing psychological distress. For example, Hamer et al. [11] utilized the 12-item General Health Questionnaire (GHQ-12) to assess the presence of psychological distress, while the study by Li et al. [13], conducted in China, measured distress with a one-item three-point rating scale.

Studies in the United States are scarce but appear to point to increased stroke risk in the presence of high distress. In community-dwelling Black and non-Hispanic White adults, age 65 and older, in Chicago, a higher distress score predicted an 18% greater risk of incident stroke after adjusting for age, race, and sex, with attenuation when further adjusted for education, stroke risk factors, chronic conditions, and medication usage [14]. In addition, previous studies in the United States have not used a standardized, validated assessment of psychological distress focused on both depression and anxiety symptoms, such as the Kessler Psychological Distress Scale, with an established cutoff and high specificity for identifying high levels of distress. A population-based study conducted in the United States utilized measures of depression, anger, and chronic burden, including health-related, relationship, and financial problems [15]. Therefore, in our current study, we aimed to ascertain the strength of the association between psychological distress and stroke, using the National Health Interview Survey (NHIS), a large national survey that is part of the Centers for Disease Control and Prevention (CDC), with a representative sample of the US population. This survey collects comprehensive health-related information through interviews, covering a wide range of health topics and demographics, including mental health and behavioral data [16], utilizing a standardized tool, that is, the Six-Item Kessler Psychological Distress Scale (K6), for the assessment of psychological distress. The K6 scale within the NHIS data demonstrates good internal consistency and psychometric validity and captures the nonspecific psychological distress construct [17].

## Materials and methods

Study population

Our study utilized data from the NHIS as the principal source of information on the health of the civilian noninstitutionalized population of the United States. The NHIS is one of the major data collection programs of the National Center for Health Statistics (NCHS) [18], which uses a multistage probability sampling design [19]. NHIS consists of cross-sectional face-to-face household interviews by trained interviewers. One adult per family is randomly selected to provide detailed health information [18,20].

Measures

We used the K6 that is incorporated in the NHIS data to measure psychological distress among US populations aged 18-64 years and older adults (≥65 years). K6 is a brief six-item standardized questionnaire used to assess for severe mental distress over a specific period of time. Individuals were asked to reflect on a 30-day period over the past 12 months when they struggled with emotional symptoms, specifically, feeling nervous, hopeless, restless and fidgety, worthless, depressed, and that everything is an effort. Ratings from 0 ("none of the time") to 4 ("all the time") result in a total maximum score of 24, with higher scores indicating greater psychological distress. The cutoff score 13 was developed to identify individuals meeting the criteria for a Diagnostic and Statistical Manual of Mental Disorders, Fourth Edition (DSM-IV) disorder with a sensitivity of 0.36 and a specificity of 0.96 as well as an established predictive validity associated with increased risk of mood and anxiety disorders among diverse populations including youth and aging populations [21,22]. The K6 measure also has an excellent internal consistency and reliability (Cronbach's alpha of 0.89) [17,23] and has been utilized in large epidemiological studies conducted on the adult US population, including those examining NHIS data, as well as other populations, such as Chinese and Canadians [1,24,25].

Statistical analysis

We used IBM SPSS Statistics for Windows, Version 26.0 (Released 2019; IBM Corp., Armonk, New York, United States) for our analyses. Measures for central tendency were employed to analyze continuous variables using the t-test and ANOVA with Bonferroni for post hoc multiple comparisons. Data presented show the mean±SEM, unless otherwise specified. For categorical variables, we used cross-tabulation with the chi-squared test to compare the observed and expected results in order to examine relationships between the various variables (data presented as percentages with a significance level (α) of 0.05). We also developed a logistic regression model, using a stepwise approach and multivariable analysis, to test the hypothesis that psychological distress (Kessler score ≥13) was associated with stroke.

In the first model, the association of psychological distress with hypertension was assessed unadjusted, whereas model 2 was minimally adjusted for age and sex. Model 3 was adjusted for age, sex, and body mass index (BMI), while model 4 was additionally adjusted for race. In model 5, the association of psychological distress with hypertension was assessed after adjusting for additional demographic factors, including education (college degree attainment), foreign-born status, poverty level, as well as marital status. In the final and complete model, we assessed the odds of stroke due to psychological stress, after adjusting for all the above factors, in addition to the major cardiovascular risk factors of stroke, including hypertension, diabetes, hypercholesterolemia, coronary heart disease, and smoking. Data were presented as odds ratio (OR) and 95% confidence interval (CI) with a significance level (α) of 0.05.

## Results

NHIS data from 2004 to 2013 included 667878 individuals; 52.7% were women, and 47.3% were men. Whites comprised 58.2%, Blacks 14.2%, Asians 6.3%, and Hispanics 21.3% of the study sample. Most did not have a college degree (75.3%), and 14.3% were below the poverty level (Table 1).

**Table 1:** Demographic characteristics and stroke risk factors among all participants of NHIS from 2004 to 2013 NHIS: National Health Interview Survey

Variable	Number	Percent
Mean age (years)±SEM	45.7±0.21	
18-44 years	333129	49.9
45-64 years	226830	34
≥65 years	107919	16.2
Sex		
Female	351812	52.7
Male	316066	47.3
Race		
White	384866	58.2
Black	94193	14.2
Hispanic	140524	21.3
Asian	41440	6.3
Educational level		
No college	489838	75.3
College	160953	24.7
Poverty level		
Above the poverty index	464745	85.7
Below the poverty index	77633	14.3
Mean BMI±SEM	27.3 ±0.01	
Normal weight	99727	36.6
Overweight (% BMI 25-29.99)	96767	35.5
Obese (% BMI ≥30)	76016	27.9
Smoking		
Never smoker	168490	58.8
Former smoker	62017	21.6
Current smoker	56264	19.6
Hypertension	88957	30.8
Diabetes	26999	9.5
Coronary heart disease	10531	3.6

**Figure 1:**
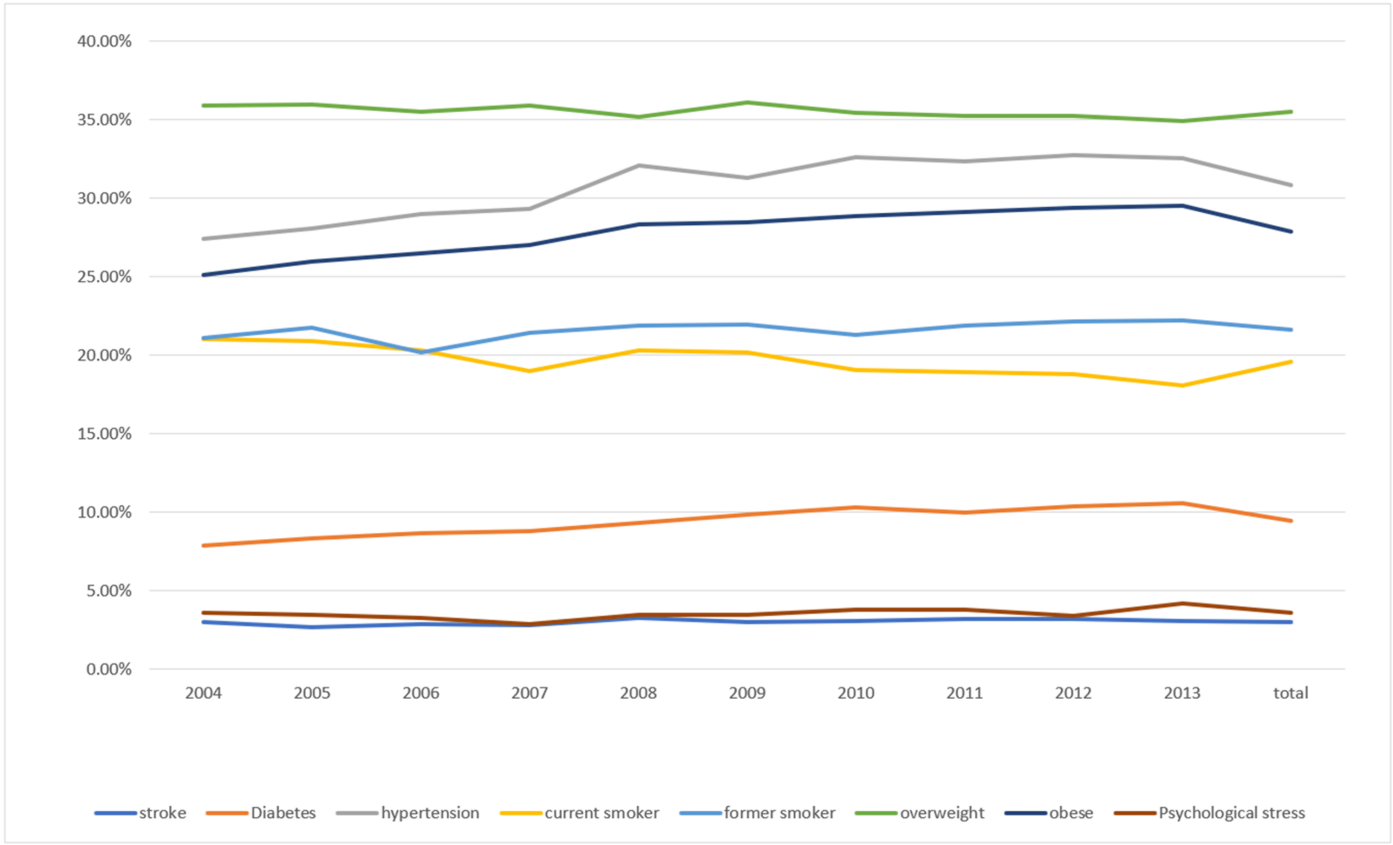
Prevalence of psychological stress, stroke, and risk factors of stroke and cardiovascular disease (2004-2013)

Psychological distress prevalence in the US adult population over the study period (2004-2013) was 3.6%, ranging from 2.9% to 4.2%, and stroke rate was 3.6% (range 2.7-3.2%) (Table 2, Figure 1). For the same time period, the hypertension rate increased from 27.4% to 32.5% (p=0.01). Diabetes rate also steadily increased from 7.9% to 10.6% (p=0.01) in the US population, as did the rate of obesity (25.1-29.5%). When combining the rates of those overweight and obese between 2004 and 2013, the rate increased from 61% to 64.5% (Table 2, Figure 1). At the same time period of the study, the rate of smoking among the US population decreased from 21% to 18.1% (p=0.01) (Table 2).

**Table 2:** Prevalence of psychological stress, stroke, and risk factors of stroke and cardiovascular disease (2004-2013)

Year	2004	2005	2006	2007	2008	2009	2010	2011	2012	2013	Total
Stroke	3%	2.7%	2.9%	2.8%	3.3%	3%	3.1%	3.2%	3.2%	3.1%	3%
Diabetes	7.9%	8.4%	8.7%	8.8%	9.3%	9.9%	10.4%	10%	10.4%	10.6%	9.5%
Hypertension	27.4%	28.1%	29%	29.3%	32.1%	31.3%	32.6%	32.4%	32.8%	32.5%	30.8%
Current smoker	21%	20.9%	20.3%	19%	20.3%	20.2%	19.1%	18.9%	18.8%	18.1%	19.6%
Former smoker	21.1%	21.8%	20.2%	21.4%	21.9%	22%	21.3%	21.9%	22.1%	22.2%	21.6%
Overweight	35.9%	36%	35.5%	35.9%	35.2%	36.1%	35.4%	35.2%	35.2%	34.9%	35.5%
Obese	25.1%	26%	26.5%	27%	28.3%	28.4%	28.9%	29.1%	29.4%	29.5%	27.9%
Psychological stress	3.6%	3.5%	3.3%	2.9%	3.5%	3.5%	3.8%	3.8%	3.4%	4.2%	3.6%

Among the 284497 NHIS participants who fully completed the Six-Item Kessler Psychological Distress Scale, the prevalence of psychological distress was higher among women compared to men (4.2% vs. 2.9%, respectively; p<0.01) (Table 3). The rates of distress were highest in Hispanics (4.1%) and Blacks (4%), as compared to Whites (3.5%) and Asians (1.9%) (p<0.01), who had the lowest distress levels. The rate of distress was reported to be highest in middle-aged adults 45-64 years of age (4.8%), lower in younger adults 18-44 years of age (3.2%), and lowest among adults 65 years of age or older (2.6%) (p<0.01). Participants who were married (55.9%) had lower rates of psychological distress (2.4%), whereas separated and divorced individuals had the highest rates of distress (8.7% and 5.7%, respectively; p<0.01) (Table 3). Those without a college degree had significantly higher distress levels (4.4%) compared to college-educated participants (1.3%) (p<0.01). High rates of distress were >3-fold higher among those below the poverty level compared to those above the poverty level (8.9% vs. 2.6%; p<0.01).

**Table 3:** Percentage of psychological stress by sociodemographic and stroke risk factors BMI: body mass index

	Psychological stress n (%)	No psychological stress n (%)	P-value
Total study population (n/%)	10218 (3.6%)	274279 (96.4%)	0.01
Age groups			
18-44 years	4199 (3.2)	128103 (96.8)	0.01
35-64 years	4534 (4.8)	90385 (95.2)	
≥65 years	1485 (2.6)	55971 (97.4)	
Sex			
Female	6622 (4.2)	152043 (95.8)	0.01
Male	3596 (2.9)	122236 (97.1)	
Race			
White	5963 (3.5)	166786 (96.5)	0.01
Black	1744 (4)	41680 (96)	
Hispanic	2067 (4.1)	48465 (95.9)	
Asian	282 (1.9)	14611 (98.1)	
Marital status			
Married	3081 (2.4)	125740 (97.6)	0.01
Never married	2719 (3.6)	72195 (96.4)	
Widowed	1069 (3.9)	26168 (96.1)	
Divorced	2442 (5.7)	40089 (94.3)	
Separated	868 (8.7)	9107 (91.3)	
Education			
No college	9179 (4.4)	199921 (95.6)	0.01
College	969 (1.3)	72594 (98.7)	
Poverty level			
Above the poverty index	5407 (2.6)	198966 (97.4)	0.01
Below the poverty index	3743 (8.9)	38253 (91.1)	
Foreign-born status			
USA born	8510 (3.7)	221276 (96.3)	0.01
Foreign born	1702 (3.1)	52718 (96.9)	
Body weight			
Mean BMI (kg/m^2^)±SD	28.7	27.3	0.01
Normal weight	2927 (3)	95451 (97)	0.01
Overweight (% BMI 25-29.99)	2800 (2.9)	92832 (97.1)	
Obese (% BMI ≥30)	4185 (5.1)	71342 (94.9)	
Smoking			
Never smoker	4020 (2.4)	162141 (97.6)	0.01
Former smoker	1941 (3.2)	59209 (96.8)	
Current smoker	4185 (7.5)	51366 (92.5)	
Hypertension			
No hypertension	5294 (2.7)	191574 (97.3)	0.01
Hypertension	4904 (5.6)	82434 (94.4)	
Diabetes			
No diabetes	8152 (3.2)	246165 (96.8)	0.01
Diabetes	1807 (6.8)	24636 (93.2)	
Hypercholesterolemia			
Normal cholesterol	1441 (2.7)	52020 (97.3)	0.01
High cholesterol	988 (4.7)	19829 (95.3)	
Coronary heart disease			
No coronary heart disease	9343 (3.4)	264823 (96.6)	0.01
Coronary heart disease	870 (8.5)	9377 (91.5)	

Unadjusted OR for stroke among psychologically distressed individuals (Table 4) was 3.1 (95% CI 2.8-3.3; p<0.01). In our minimally adjusted model (for age and sex), the OR for stroke due to psychological stress was 3.55 (95% CI 3.28-3.84; p<0.01). Further adjustment for obesity (Table 4, model 3) yielded quite similar results with an OR of 3.56 (95% CI 3.29-3.86; p<0.01). In model 4 (Table 4), after adjusting for age, sex, BMI, and race, the OR of stroke due to psychological distress remained similar to the previous two models (OR 3.58; 95% CI 3.30-3.3.88; p<0.01). As compared to Whites, Blacks had a 57% increased odds of stroke. Hispanics (OR 0.92; 95% CI 0.85-0.99; p=0.03) and Asians (OR 0.72; 95% CI 0.62-0.82; p=0.01) had a lower risk of stroke compared to Whites (Table 4, model 4). 

**Table 4:** Associations between psychological distress and stroke after adjusting for demographic and stroke risk factors BMI: body mass index

Logistic regression model	Odds ratio	95% CI	P-value
Model 1 (unadjusted)			
Psychological distress	3.1	2.88-3.34	0.01
Model 2 (minimally adjusted for age and sex)			
Psychological distress	3.55	3.29-3.84	0.01
Age 18-44	1.0 (reference)		
Age 45-64	5.6	5.15-6.09	0.01
Age ≥65	18.31	16.90-19.83	0.01
Male sex	1 (reference)		
Female sex	1	0.95-1.04	0.99
Model 3 (adjusted for age, sex, and obesity)			
Psychological distress	3.56	3.29-3.86	0.01
Age 18-44	1.0 (reference)		
Age 45-64	5.48	5.03-5.97	0.01
Age ≥65	18.14	16.71-19.70	0.01
Male sex	1 (reference)		
Female sex	0.99	0.94-1.03	0.98
BMI (kg/m^2^)			
Normal (BMI 18.5-24.99)	1.0 (reference)		
Overweight (BMI 25-29.99)	1.08	1.02-1.14	0.01
Obese (BMI ≥25)	1.35	1.27-1.43	0.01
Model 4 (adjusted for age, sex, obesity, and race)			
Psychological distress	3.58	3.30-3.88	0.01
Age 18-44	1.0 (reference)		
Age 45-64	5.44	4.99-5.93	0.01
Age ≥65	18.29	16.83-19.89	0.01
Male sex	1.0 (reference)		
Female sex	0.98	0.94-1.03	0.59
BMI (kg/m^2^)			
Normal (BMI 18.5-24.99)	1.0 (reference)		
Overweight (BMI 25-29.99)	1.05	0.99-1.11	0.05
Obese (BMI ≥25)	1.28	1.20-1.35	0.01
Race			
White	1.0 (reference)		
Black	1.57	1.48-1.66	0.01
Hispanic	0.92	0.85-0.99	0.03
Asian	0.72	0.62-0.82	0.01

Foreign-born individuals had 32% lower odds of stroke due to psychological stress (Table 5). The frequency of foreign-born status among various ethnic groups was significantly different: N (%) for Whites 19025 (5%), for Blacks 10118 (10.8%), for Hispanics 86265 (61.8%), and for Asians 32013 (78.2%) (p<0.01). After adjusting for foreign-born status, the odds of stroke due to psychological stress among Hispanics (OR 0.97; 95% CI 0.88-1.07; p=0.63) and Asians (OR 0.89; 95% CI 0.75-1.05; p=0.1) were not significantly different compared to Whites (Table 5).

**Table 5:** Associations between psychological distress and stroke after adjusting for additional demographic factors Adjusted model controlling for demographic and stroke risk factors: age, sex, race, education, foreign-born status, poverty level, marital status, and obesity BMI: body mass index

Logistic regression model	Odds ratio	95% CI	P-value
Model 5			
Psychological distress	2.9	2.66-3.17	0.01
Age 18-44	1.0 (reference)		
Age 45-64	4.96	4.51-5.45	0.01
Age ≥65	14.64	13.29-16.13	0.01
Male sex	1 (reference)		
Female sex	0.91	0.86- 0.96	0.01
BMI (kg/m^2^)			
Normal (BMI 18.5-24.99)	1.0 (reference)		
Overweight (BMI 25-29.99)	1.07	1.00-1.14	0.03
Obese (BMI ≥25)	1.23	1.15-1.31	0.01
Race			
White	1 (reference)		
Black	1.39	1.31-1.49	0.01
Asian	0.89	0.75-1.05	0.17
Hispanic	0.97	0.88-1.07	0.63
No college degree	1 (reference)		
College degree (yes)	0.61	0.57-0.66	0.01
US born	1 (reference)		
Foreign born	0.68	0.62-0.75	0.01
Above the poverty level	1 (reference)		
Below the poverty level	1.82	1.70-1.94	0.01
Marital status			
Married	1 (reference)		
Never married	1.31	1.20-1.44	0.01
Widowed	1.69	1.53-1.88	0.01
Divorced	1.54	1.39-1.70	0.01
Separated	1.55	1.34-1.79	0.01

As shown in Table 5 (model 5), we further adjusted for educational level, poverty level, and foreign-born status as well as marital status. These adjustments attenuated the odds of stroke due to psychological stress, compared to previous models (OR 2.9; 95% CI 2.66-3.17; p<0.01). In this model, the odds of stroke among Blacks, compared to Whites, by psychological stress remained high, though attenuated compared to the previous model from 57% to 39% (OR 1.39; 95% CI 1.31-1.49; p< 0.01). See Tables 4-5.

In the final logistic regression model, we added the cardiovascular risk factors for stroke, including hypertension, diabetes, hypercholesterolemia, coronary heart disease, and smoking. Despite adjusting for age, sex, race, obesity, and other sociodemographic factors, the odds of stroke due to cardiovascular risk factors remains high in this full model (Table 6).

**Table 6:** Associations between psychological distress and stroke after adjusting for additional stroke risk factors Adjusted model controlling for demographic and stroke risk factors: age, sex, race, education, foreign-born status, poverty level and marital status, obesity, hypertension, diabetes, high cholesterol, coronary heart disease, and smoking BMI: body mass index

Logistic regression model	Odds ratio	95% CI	P-value
Model 6			
Psychological distress	2.17	1.81-2.60	0.01
Age 18-44	1.0 (reference)		
Age 45-64	3.26	2.68-3.97	0.01
Age ≥65	6.23	5.05-7.68	0.01
Male sex	1.0 (reference)		
Female sex	1.1	0.98- 1.22	0.07
BMI (kg/m^2^)			
Normal (BMI 18.5-24.99)	1.0 (reference)		
Overweight (BMI 25-29.99)	0.92	0.81-1.04	0.2
Obese (BMI ≥25)	0.86	0.75-0.98	0.03
Race			
White	1 (reference)		
Black	1.21	1.06-1.38	0.01
Asian	0.98	0.72-1.32	0.9
Hispanic	1	0.83-1.20	0.97
No college degree	1.0 (reference)		
College degree (yes)	0.67	0.58-0.78	0.01
US born	1.0 (reference)		
Foreign born	0.81	0.67-0.98	0.03
Above the poverty level	1.0 (reference)		
Below the poverty level	1.62	1.43-1.84	0.01
Marital status			
Married	1 (reference)		
Never married	1.18	0.98-1.41	0.07
Widowed	1.43	1.17-1.75	0.01
Divorced	1.26	1.04-1.53	0.02
Separated	1.24	0.93-1.65	0.14
Smoking			
Never smoker	1 (reference)		
Former smoker	1.29	1.15-1.45	0.01
Current smoker	1.42	1.23-1.63	0.01
Hypertension	2.54	2.24-2.87	0.01
Diabetes	1.68	1.49-1.88	0.01
High cholesterol	1.44	1.29-1.60	0.01
Coronary heart disease	3.27	2.87-3.71	0.01

Hypertension more than doubled the odds of stroke with an OR of 2.54 (95% CI 2.24-2.87; p<0.01). Diabetes increased the odds of stroke by 68% with an OR of 1.68 (95% CI 1.49-1.88; p<0.01). Hypercholesterolemia was also strongly associated with stroke risk (OR 1.44; 95% CI 1.29-1.60; p<0.01). Coronary heart disease, which is a stroke equivalent, more than tripled the odds of stroke (OR 3.27; 95% CI 2.87-3.71; p<0.01). Compared to never smoking, current smokers and former smokers had an OR of 1.29 (95% CI 1.15-1.45; p<0.01) and 1.42 (95% CI 1.23-1.63; p<0.01), respectively.

In models 4 and 5 (Table 4, Table 5), obesity (BMI ≥25) conferred a significant increase in odds of stroke due to psychological distress (OR 1.28, 95% CI 1.20-1.35, and p<0.01 and OR 1.23, 95% CI 1.15-1.31, and p<0.01, respectively). However, after adjusting for cerebrovascular risk factors (model 6), including diabetes, hypertension, high cholesterol, coronary heart disease, and smoking, obesity had a favorable effect on the odds of stroke risk due to psychological distress, with a 14% decreased odds of stroke in obese compared to non-obese individuals (OR 0.86; 95% CI 0.75-0.98; p=0.03).

Finally, after adjusting for age, sex, race, obesity, and other sociodemographic risk factors, as well as cardiovascular risk factors, the odds of stroke conferred by psychological distress, although attenuated, remained significantly high, more than double among psychologically distressed individuals (OR 2.17; 95% CI 1.81-2.60).

## Discussion

Our study indicates a strong association between psychological distress and stroke, with more than double the odds of stroke among psychologically stressed individuals compared to those without psychological distress. This is the first study to examine this relationship in a large sample of participants representative of the adult US population over a decade, utilizing the K6, a validated, standardized measure with high specificity for identifying psychological distress and with an excellent internal consistency and reliability (Cronbach's alpha of 0.89) [17,23].

Our findings of a significant association between psychological distress and increased stroke risk are consistent with previous reports, including the findings in large samples in Australia and the United Kingdom [26,27]. The previous studies include a prospective cohort in the United Kingdom, which showed that increased psychological distress is significantly associated with stroke risk [27]. Psychological distress was also found to be a predictor of fatal ischemic stroke in men in Australia [26]. In another large Australian sample of participants aged 45 years and over, heart disease, stroke, and diabetes were all significantly associated with psychological distress on the 10-Item Kessler Psychological Distress Scale [28].

In the Multi-Ethnic Study of Atherosclerosis (MESA), a longitudinal cohort study that examines risk factors for clinical and subclinical cardiovascular disease [29], there was an increased risk of incident stroke or transient ischemic attack with higher levels of chronic stress burden, hostility, and depression in multiethnic middle-aged and older adults aged 45-84 years and free of clinical cardiovascular disease at baseline [30]. In this study, conducted at six US sites, chronic stress conferred a 59% increased risk (OR 1.59; 95% CI 1.11-2.27) [30].

The MESA study assessed chronic stress, depressive symptoms, trait anger, and hostility using standard questionnaires, with the primary outcome focused on clinically adjudicated incident stroke or transient ischemic attacks during a median follow-up of 8.5 years [29]. While this study is longitudinal, as opposed to our current study, which is cross-sectional, with results supporting our hypothesis and our study findings, the MESA study did not examine psychological distress with the validated K6 scale that is robust and practical, with great applicability to large and diverse population screening for psychological distress [22-25].

An interesting finding in our analyses is that obesity conferred a significant risk of stroke due to psychological distress, in models unadjusted for cardiovascular disease risk factors [3,5], such as hypertension, diabetes, coronary heart disease, hypercholesterolemia, and smoking. However, after controlling for these cerebrovascular risk factors, obesity in fact offered a 14% decreased risk of stroke (Table 6, model 6). This important finding from our study not only indicates that the risk of stroke with obesity is likely mediated by those abovementioned cardiovascular risk factors but is also consistent with the concept of the obesity paradox [30-34], that is, the observation that while obesity is generally implicated in multiple health conditions, in certain disorders or certain patient populations, obese individuals might fare better, compared to those with normal weight, as was observed in our study (Table 6). For example, in diseases such as heart failure, coronary artery disease, chronic kidney disease, and those with dialysis, obese patients might have better outcomes compared to normal body weight or underweight individuals [35-38]. Work by our group and others supports the notion of the obesity paradox, whereby better outcomes were noted in obese patients undergoing percutaneous cardiovascular interventions for coronary artery disease, with strong disparity between different sexes and races [30-31]. Furthermore, another study by our group showed a graded increase in bone mineral density in the obese population, thus offering fracture protection. In this study, we also documented disparity where the favorable effect of obesity on bone mass was observed in Whites and Hispanics, but not in the Black population [39]. These previous findings and the results of our current study challenge the dogma that obesity is universally associated with worse health outcomes [40-43]. Our current findings also provide further support to the new classification of obesity into preclinical and clinical categories [44], which was published in January 2025 and endorsed by 79 international, regional, and national scientific societies, including the World Obesity Federation (WOF), with the main message that not all obesity is necessarily harmful.

Our study is conducted on a large representative sample of the adult US population with 284497 individuals, using K6, a standardized, validated, and, importantly, brief and practical tool to assess psychological distress in various clinical settings. Our findings provide evidence that psychological distress is a potentially modifiable risk factor for stroke that is easily identifiable and that confers a 117% increased odds of stroke with psychological distress after adjusting for demographic and specific cardiovascular risk factors. These findings have potentially important implications for public health given that stroke is number 4 among the top 10 causes of death in the United States [1] and is associated morbidity and healthcare cost that continue to rise [2], particularly with advanced therapeutic intervention and standardization of stroke care that led to increased stroke survival. Stroke certainly meets the established criteria for disease prevention [45], and psychological distress appears to be an attractive target for stroke prevention based on the results of our study and previous research, based on the ease of use of K6 in clinical practice with various populations, and in the context of mounting evidence showing the effectiveness of interventions on stroke risk factors.

Specifically, several studies showed that emotional social support (ESS) is effective in the prevention of the incidence of hard cardiovascular disease [46-50]. Empirically supported interventions, such as cognitive behavioral therapy (CBT), can significantly reduce distress, including depression and anxiety, by identifying and correcting maladaptive thoughts and behaviors [50]. CBT-based interventions and mindfulness-based stress reduction (MBSR) have been effective in treating patients with chronic illness [51-54]. Behavioral interventions aimed at reducing psychological distress have been shown to improve hypertension, cholesterol levels [55,56], as well as blood glucose levels [51]. CBT-based intervention in patients with coronary heart disease was shown to be effective in reducing stress symptoms and also BMI [53].

In our study, after controlling for hypertension, diabetes, and other significant stroke risk factors, the odds of stroke in those with psychological distress is more than double. Therefore, interventions addressing psychological distress, such as ESS, MBSR, and CBT, will further reduce the risk of stroke, either directly or by mitigating the deleterious effects of hypertension, diabetes, and other vascular diseases.

Putative mechanisms of stroke associated with chronic psychological stress

**Figure 2:**
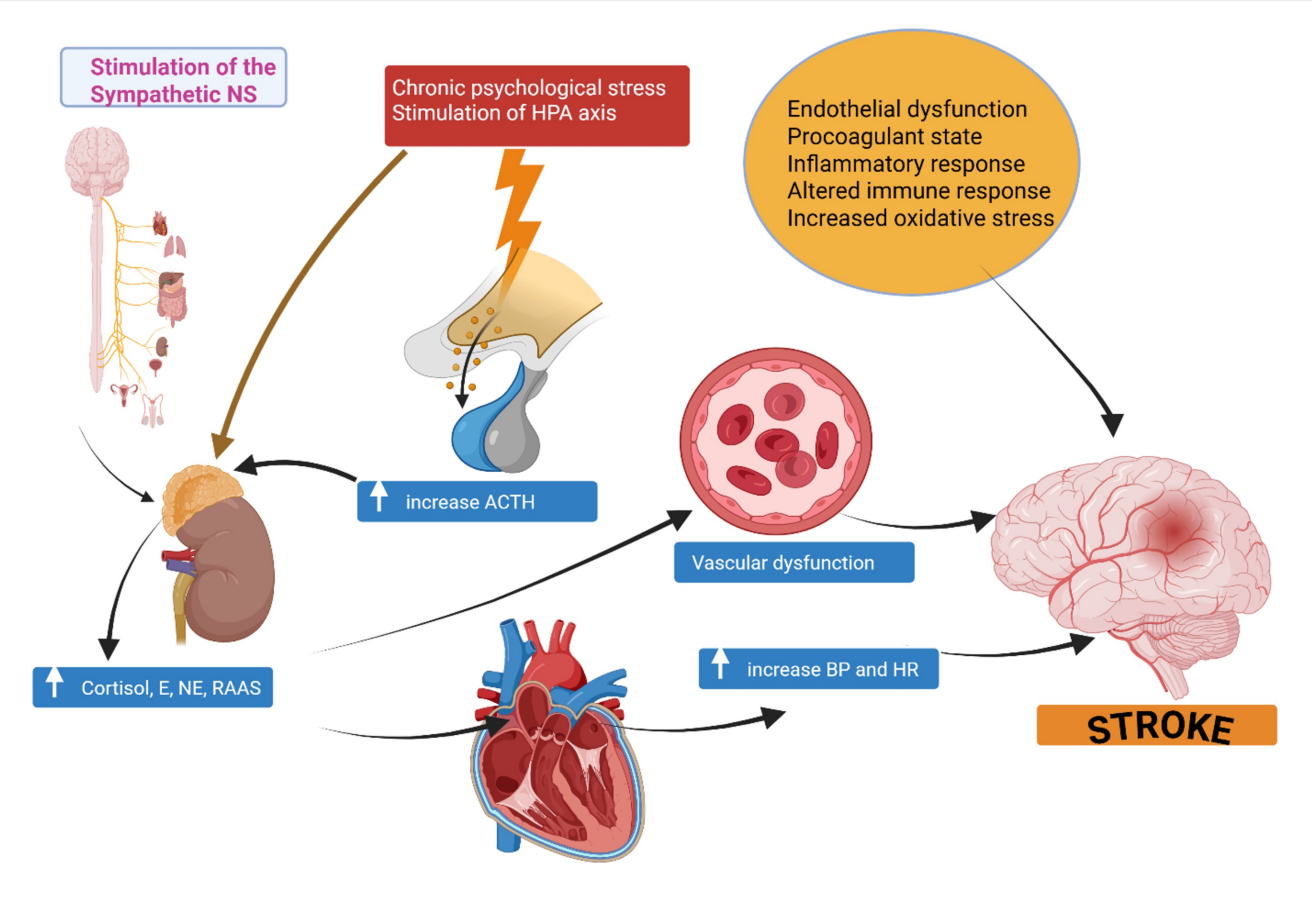
Putative mechanisms of stroke associated with chronic psychological stress NS: nervous system; HPA: hypothalamic-pituitary-adrenal; ACTH: adrenocortical hormone; E: epinephrine; NE: norepinephrine; BP: blood pressure; HR: heart rate; RAAS: renin-angiotensin-aldosterone system

Several mechanisms have been postulated to explain the relationship between psychological stress and cardiovascular disease [6,55-63]. These include neurohormonal activation with the stimulation of the hypothalamic-pituitary-adrenal axis as well as the sympathetic nervous system, leading collectively to increased epinephrine and cortisol and the stimulation of the renin-angiotensin-aldosterone system (RASS) (Figure 2). These result in an increase in blood pressure, heart rate, and blood viscosity with a procoagulant state that would promote a thrombotic milieu and increase the risk of stroke. Furthermore, altered immune response, increased oxidative stress, as well as endothelial dysfunction [62] further promote thrombotic events [6,55-63].

In addition to the above-proposed biological mechanisms, the relationship between distress and stroke may be driven by poor treatment adherence among distressed patients. Treatment adherence is affected by the motivational and behavioral disturbances associated with psychological distress. There is ample evidence from studies showing high rates of nonadherence in depressed patients [61], and this extends to patients who are not clinically depressed but struggle with symptoms of distress [62,63]. CBT-based interventions can correct misconceptions about medicines and illness and thus improve medication adherence when managing hypertension, diabetes, and other risks. These approaches may also target other health behaviors, such as smoking, exercise, and diet. In turn, improved health behaviors will likely lead to lowered odds of stroke [63,6].

While more research is needed to understand the drivers of the relationship between psychological distress and stroke, there appear to be several pathways that present clear targets for patient care. Successful interventions may be particularly impactful given the unfavorable change in stroke mortality and the aging US population, where stroke death rates have declined more slowly, stalled, or even reversed among some sub-populations in recent years [64].

Study limitations

The cross-sectional nature and the self-reporting of stroke and other variables included in this study do not provide information about causality or whether psychological stress prospectively increases risks leading to stroke or, conversely, reflects post-stroke distress and depression; however, data available from prospective studies such as the MESA study [29,64] suggest the former.

Other limitations are related to the K6 scale, used in our study, including the detection of nonspecific psychological symptoms and its sensitivity. While having high specificity to identify general distress, sensitivity when using a 13 cutoff may result in false negatives, missing those with actual distress.

## Conclusions

Utilizing a decade worth of data from a large, nationally representative US sample (NHIS) with a validated measure of psychological distress (K6), our study findings indicate that individuals reporting psychological distress had more than twice increased odds of stroke compared to those without distress. 

This study of the general US population indicates a strong association between psychological distress and stroke after adjusting for age, sex, race, obesity, and other cardiovascular risk factors for stroke, including hypertension, diabetes, hypercholesterolemia, coronary heart disease, and smoking, as well as demographic factors, such as education, poverty level, marital status, and foreign-born status. These results indicate that psychological distress, easily identifiable in clinical practice with a six-item scale, is a potentially modifiable risk factor for stroke, which can be targeted with effective interventions to reduce stress and, thereby, reduce stroke and other cardiovascular diseases.
